# Balint in the time of COVID-19: participant and facilitator experience of virtual Balint groups compared with in-person

**DOI:** 10.1192/bjo.2021.432

**Published:** 2021-06-18

**Authors:** Sheliza Samnani, Masud Awal

**Affiliations:** Birmingham and Solihull Mental Health Foundation Trust

## Abstract

**Aims:**

Our Trust increased Balint group provision, relocating virtually for psychiatry doctors to explore the emotional impact of clinical practice and doctor-patient relationships, during unfamiliar challenges of the pandemic. This unique context allowed comparison of multiple virtual and face-to-face (F2F) Balint-type group experiences for participants and facilitators.

**Method:**

In March 2020, existing core trainee (CT) year 1 and 2, higher trainee (ST) and consultant Balint groups became virtual, with new CT3 and Speciality Doctor and Associate Specialist (SAS) virtual Balint groups established.

All 57 participants and 5 facilitators were sent an anonymous electronic survey to retrospectively rate virtual Balint (March-August 2020) and their preceding F2F Balint group (suggesting September 2019-February 2020) experience.

**Result:**

The response rate was 89% for participants (51 respondents) and 100% for facilitators (5 respondents).

For group participants, 90% (virtual) and 78% (F2F) agreed or strongly agreed that Balint group provided an opportunity to explore challenging aspects of clinical work. 76% (virtual) and 71% (F2F) agreed or strongly agreed that it made them feel more supported. Almost 50% agreed or strongly agreed that virtual and F2F Balint group helped work feel less stressful. Both ratings and free-text feedback emphasised virtual Balint attendance being easier.

Facilitators rated virtual and F2F formats similarly highly with regards to exploring difficult doctor-patient interactions, richness of discussions and their enjoyment. Facilitators felt virtual attendance was easier but more draining, with more difficult adherence to Balint group etiquette and boundaries.

82% of participants and 75% of facilitators agreed or strongly agreed that virtual format made them more likely to attend future Balint groups. The rich pool of free-text comments received were predominantly positive, whilst noting challenges during virtual Balint in remaining present, with more distractions (for participants) and additional difficulty accessing group dynamics (for facilitators).

**Conclusion:**

Participant and facilitator responses indicate Balint-type groups being professionally and clinically beneficial across different psychiatrist grades, and promoting clinician wellbeing when both F2F and virtual during pandemic-related restrictions. Facilitator ratings (unlike participants) suggested specific virtual process challenges such as feeling more drained, perhaps in part due to technical application issues around this emerging format.

Both participants and facilitators reported attendance being easier when virtual. Although some suggested returning to F2F post-COVID, more preferred to continue virtually or utilise a blended format. This was particularly for non-CT groups where geographical challenges (e.g. region-wide ST Balint) or competing clinical demands (e.g. consultant/SAS Balint) made regular commitment and attendance more difficult.

